# Pharmaceutical Expenditure and Consumption of Recommended Drugs to Avoid in Italy

**DOI:** 10.1001/jamanetworkopen.2024.46237

**Published:** 2024-11-20

**Authors:** Filomena Fortinguerra, Benedetta Bellini, Antonietta Colatrella, Francesco Trotta

**Affiliations:** 1 Italian Medicines Agency, Rome, Italy

## Abstract

This quality improvement study examines public pharmaceutical expenditure and consumption of drugs from the 2023 annual list of recommended drugs to avoid released by *Prescrire*.

## Introduction

Each year, the independent French drug bulletin *Prescrire* releases a list of so-called drugs to avoid, a list of medicines that entered the European market between 2010 and 2022 and are considered to be more harmful than beneficial for patients according to relative effectiveness assessments based on the rigorous methods of evidence-based medicine.^1^ The aim of this drugs-to-avoid list is to provide a simple tool for prescribers about the real therapeutic value of new medicines and help to avoid unnecessarily hazardous prescriptions. It can also be used to evaluate newly authorized medicines over time and across therapeutic categories and to assess the quality of drug prescription.^2,3,4^ We conducted a descriptive analysis on expenditure and consumption of drugs to avoid listed by *Prescrire International* in 2023 and reimbursed in Italy during 2022.

## Methods

For this quality improvement study, no review or approval by an institutional review board or ethics committee was needed because this is a descriptive study based on retrospective data routinely collected and kept properly anonymized within administrative health care databases, with no need to obtain an informed consent at the time of original data collection. We followed the Standards for Quality Improvement Reporting Excellence (SQUIRE) reporting guideline.

The drugs-to-avoid list published in April 2023 issue of *Prescrire International*^5^ were categorized by Anatomical Therapeutic Chemical (ATC) group and level.^6^ For all drugs reimbursed in Italy with the same active ingredient, formulation and dosage as reported in the list, the national data on expenditure (in euros), and consumption (number of defined daily doses per 1000 inhabitants per day)^6^ in 2022 were calculated through national pharmaceutical databases and compared with those of overall drugs to avoid, drugs to avoid with the same ATC level 1 group, and drugs reimbursed in Italy with the same ATC level 3 classification, given that they can be considered as their therapeutic alternatives.

## Results

Of 115 drugs to avoid, 56 drugs (48.7%) belonging to 11 ATC groups were reimbursed in Italy in 2022 (Figure; eTable 1 in Supplement 1). They were responsible for an expenditure of €1.15 billion (approximately US $1.25 billion), corresponding to 4.9% of the total expenditure. The consumption of these drugs accounted for 86.2 defined daily doses per 1000 inhabitants per day, corresponding to the 6.9% of the consumption of all reimbursed drugs (Table). In all, 15 drugs covered 75% of expenditure and 80% of consumption of the overall drugs-to-avoid list: 5 cardiovascular agents, such as 3 antihypertensives (olmesartan alone or combined with amlodipine or hydrochlorothiazide), ranolazine, and fenofibrate; 3 glucose-lowering drugs for type 2 diabetes (linagliptin, sitagliptin, and sitagliptin/metformin); 4 antidepressants (escitalopram, duloxetine, venlafaxine, and citalopram); 2 antineoplastic and immunomodulating agents (natalizumab and nintedanib); and denosumab, a monoclonal antibody for osteoporosis. Olmesartan alone or in combination accounted for up to 28.6% of the consumption of the negative list (and up to 39.0% of expenditure) compared with their therapeutic alternatives (drugs with the same ATC level 3).

**Figure.**
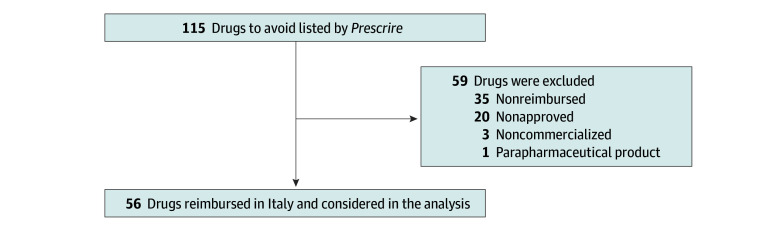
Drug Selection Based on *Prescrire International*’s 2023 List of Drugs to Avoid

**Table.** Expenditure and Consumption of Reimbursed Drugs from the *Prescrire International* 2023 List of Drugs to Avoid, by ATC Level in Italy, 2022

Level 1 ATC group, active ingredient	Expenditure				Consumption			
	€ (US$)	%			DDD per 1000 inhabitants, No.	%		
		By level 1 ATC group	Overall	Among all level 3 ATCs reimbursed		By level 1 ATC group	Overall	Among all level 3 ATCs reimbursed
Overall (N = 56)	1 150 925 647 (1 254 508 955)	100	100	4.92	86.21	100	100	6.89
**C: cardiovascular system **								
Overall (n = 9)	380 695 137 (414 957 699)	100	33.08	11.19^a^	42.60	100	49.41	8.44^a^
C09CA08: olmesartan	113 132 397 (123 314 313)	29.72	9.83	39.01	16.74	39.29	19.41	28.61
C09DB02: olmesartan/amlodipine	85 779 315 (93 499 453)	22.53	7.45	19.74	9.87	23.17	11.45	22.71
C09DA08: olmesartan/hydrochlorothiazide	74 484 590 (81 188 203)	19.57	6.47	17.14	10.41	24.45	12.08	23.96
C01EB18: ranolazine	72 071 049 (78 557 443)	18.93	6.26	NA	1.24	2.92	1.44	NA
C10AB05: fenofibrate	22 507 075 (24 532 712)	5.91	1.96	2.58	2.75	6.44	3.18	2.84
Others (n = 4)^b^	12 720 710 (13 865 574)	3.34	1.11	NA	1.59	3.73	1.84	NA
**L: antineoplastic and immunomodulating agents**								
Overall (n = 7)	228 000 974 (248 521 062)	100	19.81	3.29^a^	0.16	100	0.18	0.85^a^
L04AA23: natalizumab	111 489 597 (121 523 661)	48.90	9.69	NA	0.10	66.29	0.12	NA
L01EX09: nintedanib^c^	93 824 798 (102 269 030)	41.15	8.15	NA	0.05	30.39	0.06	NA
Others (n = 5)^b^	22 686 579 (24 728 371)	9.95	1.97	NA	0.01	3.31	0.01	NA
**N: nervous system **								
Overall (n = 11)	199 729 498 (217 705 153)	100	17.35	10.10^a^	21.57	100	25.03	22.06^a^
N06AB10: escitalopram	57 227 558 (62 378 038)	28.65	4.97	14.14	7.75	35.91	8.99	17.06
N06AX21: duloxetine	48 489 999 (52 854 099)	24.28	4.21	11.98	3.43	15.90	3.98	7.55
N06AX16: venlafaxine	48 234 250 (52 575 333)	24.15	4.19	11.92	3.89	18.04	4.52	8.57
N06AB04: citalopram	23 546 663 (25 665 863)	11.79	2.05	5.82	4.12	19.10	4.78	9.07
Others (n = 7)^b^	22 231 029 (24 231 822)	11.13	1.93	NA	2.38	11.04	2.76	NA
**M: musculoskeletal system **								
Overall (n = 10)	167 075 466 (182 112 258)	100	14.52	28.50^a^	14.37	100	16.67	31.68^a^
M05BX04: denosumab	82 475 288 (89 898 064)	49.36	7.17	39.32	4.50	31.32	5.22	33.18
Others (n = 9)^b^	84 600 178 (92 214 194)	50.64	7.35	NA	9.87	68.68	11.45	NA
**A: alimentary tract and metabolism **								
Overall (n = 12)	165 829 275 (180 753 910)	100	14.41	5.09^a^	6.67	100	7.74	2.10^a^
A10BH05: linagliptin	46 421 471 (50 599 403)	27.99	4.03	4.93	1.69	25.39	1.96	3.19
A10BH01: sitagliptin	31 969 124 (34 846 345)	19.28	2.78	3.40	1.43	21.49	1.66	2.70
A10BD07: sitagliptin/metformin	29 823 351 (32 507 453)	17.98	2.59	3.17	1.40	20.95	1.62	2.63
Others (n = 9)^b^	57 615 329 (62 800 709)	34.74	5.01	NA	2.15	32.17	2.49	NA
**Other groups**								
G: genitourinary system, sex hormones (n = 2)	7 946 388 (8 661 563)	100	0.69	2.13^a^	0.80	100	0.93	1.79^a^
J: anti-infectives for systemic use (n = 1)	1 091 497 (1 189 732)	100	0.09	0.04^a^	0.02	100	0.03	0.11^a^
R: respiratory system (n = 2)	254 759 (277 687)	100	0.02	0.02^a^	0.01	100	0.01	<0.01^a^
D: dermatologicals (n = 2)	302 653 (329 892)	100	0.03	0.12^a^	0.01	100	0.01	0.06^a^

Abbreviations: ATC, Anatomical Therapeutic Chemical; DDD, defined daily doses; NA, not applicable.

^a^ Calculated on ATC level 1 group; for natalizumab, nintedanib, and ranolazine, it was not possible to make comparisons by ATC level 3, given their specific therapeutic indications.

^b^ Only drugs with an expenditure greater than €20 million (US $22 million) were shown as single active ingredient.

^c^ Nintedanib is mentioned twice in the 2023 drugs to avoid list: in lung cancer (ATC L) and idiopathic pulmonary fibrosis (ATC R), but it has been counted as 1 drug.

## Discussion

To our knowledge, this quality improvement study is the first study performed in Italy on national expenditure and consumption of the *Prescrire* drugs-to-avoid list. Our study showed that almost half of the drugs that *Prescrire* recommends avoiding were reimbursed in Italy, but only a few of them were widely prescribed (ie, olmesartan) compared with their therapeutic alternatives, resulting in a limited impact on public pharmaceutical expenditure and consumption. Despite *Prescrire*’s recommendations, no shifts in reimbursement or regulatory status were observed for these medicines in Italy nor in 2 non-European countries (Canada and Australia) where similar studies were performed.^2,3,4^ This may be because some tools were in place in Italy, such as therapeutic plans or notes limiting drug reimbursement or prescription, with the aim to promote the optimal use of reimbursed medicines. A limitation of this study was that the drug expenditure and consumption could be overestimated, such as if the medicine was dispensed but not actually used by the patient.

## Notes

Supplement 1. **eTable.** Status of *Prescrire*’s List of Drugs to Avoid 2023 in Italy

Supplement 2.
Data Sharing Statement

## References

[1] L’année 2009 du médicament: trop peu de progrès pour soigner et trop de régressions. La Revue Prescrire. 2010;30(136):136-142.

[2] Vitry A, Mintzes B. “Drugs to avoid” to improve quality use of medicines: how is Australia faring? J Pharm Policy Pract. 2021;14(1):60. doi:10.1186/s40545-021-00346-3 34256874 PMC8278758

[3] Mintzes B, Vitry A. ‘Drugs to avoid’: can we improve prescribing appropriateness? Drug Ther Bull. 2021;59(11):162. doi:10.1136/dtb.2021.000029 34711643

[4] Lexchin J. Canadian status of “drugs to avoid” in 2017: a descriptive analysis. CMAJ Open. 2018;6(3):E430-E435. doi:10.9778/cmajo.20180049 30266781 PMC6182123

[5] Towards better patient care: drugs to avoid in 2023. Prescrire Int. 2023;32(245):50-1–50-11

[6] WHO Collaborating Centre for Drug Statistics Methodology. Guidelines for ATC classification and DDD assignment 2022. Oslo, Norway, 2023. Updated September 23, 2023. Accessed September 27, 2024. https://www.whocc.no/atc_ddd_index/

