# Supplementary material for: Pharmaceutical Expenditure and Consumption of Recommended Drugs to Avoid in Italy
Source: JAMA Netw Open. 2024 Nov 20;7(11):e2446237. doi: 10.1001/jamanetworkopen.2024.46237 (PMC11579788; doi:10.1001/jamanetworkopen.2024.46237)
Supplement: Supplement 1. — eTable. Status of Prescrire’s List of Drugs to Avoid 2023 in Italy [file jamanetwopen-e2446237-s001.pdf]

Supplemental Online Content

Fortinguerra F, Bellini B, Colatrella A, Trotta F. Pharmaceutical expenditure and consumption of recommended drugs to avoid in Italy. *JAMA Netw Open*. 2024;7(11):e2446237. doi:10.1001/jamanetworkopen.2024.46237

eTable. Status of *Prescrire*'s List of Drugs to Avoid 2023 in Italy

This supplemental material has been provided by the authors to give readers additional information about their work.

© 2024 Fortinguerra F et al. *JAMA Network Open.*

eTable 1. Status of *Prescrire*'s list of "Drugs to avoid" 2023 in Italy (updated May 2023)[a]

ATC V level	Therapeutic area, Active ingredient	Status in 2022[a]
	**Oncology, Haematology, Transplantation**	
B01AX01	Defibrotide	non-reimbursed
L01EX09	Nintedanib[b]	reimbursed
L03AX15	Mifamurtide	reimbursed
L01XH03	Panobinostat	reimbursed but non commercialized
B03XA05	Roxadustat	reimbursed but non commercialized
L01CX01	Trabectedin	reimbursed
L01EX04	Vandetanib	reimbursed
L01CA05	Vinflunine	reimbursed
	**Cardiology**	
C09XA02	Aliskirèn	reimbursed
C10AB02	Bézafibrate	reimbursed
C10AB08	Ciprofibrate	non-approved
C10AB05	Fenofibrate	reimbursed
C01BD07	Dronedarone	reimbursed
C01EB17	Ivabradine	reimbursed
C01DX16	Nicorandil	non-reimbursed
C09CA08	Olmesartan	reimbursed
C09DA08	Olmesartan/hydrochlorothiazide	reimbursed
C09DB02	Olmesartan/amlodipine	reimbursed
C01EB18	Ranolazine	reimbursed
C01BG11	Vernakalant	non-reimbursed
C01EB15	Trimétazidine	non-reimbursed
	**Dermatology, Allergy**	
G04CB01	Finasteride 1 mg	non-approved
D11AH02	Pimecrolimus (topical)	reimbursed
R06AD07	Mequitazine	non-reimbursed
D11AH01	Tacrolimus (topical)	reimbursed
R06AD02	Prométhazine (injectable)	reimbursed
V01AA08	Peanut protein	non-approved
	**Diabetes, Nutrition**	
A10BH04	Alogliptin	reimbursed
A10BD13	Alogliptin/metformin	reimbursed
A10BH05	Linagliptin	reimbursed
A10BD11	Linagliptin/metformin	reimbursed
A10BH03	Saxagliptin	reimbursed
A10BD10	Saxagliptin/metformin	reimbursed
A10BH01	Sitagliptin	reimbursed
A10BD07	Sitagliptin/metformin	reimbursed
A10BH02	Vildagliptin	reimbursed
A10BD08	Vildagliptin/metformin	reimbursed
A10BG03	Pioglitazone	reimbursed
A08AA62	Bupropion + naltrexone	non-reimbursed
A08AB01	Orlistat	non-reimbursed
	**Pain, Rheumatology**	
M01AB16	Acéclofénac (oral)	reimbursed
M01AB05	Diclofenac (oral)	reimbursed
M01AH01	Celecoxib	reimbursed
M01AH05	Etoricoxib	reimbursed
M01AH04	Parecoxib	non-reimbursed
M02AB01	Capsaicin (patch)	non-reimbursed
M04AC01	Colchicine + opium powder + tiemonium	reimbursed
M05BX04	Denosumab 60 mg	reimbursed
M01AX21	Diacerein	non-reimbursed
M01AX05	Glucosamine	non-reimbursed
M02AA10	Ketoprofen (gel)	non-reimbursed
M01AC06	Meloxicam	reimbursed
M01AC01	Piroxicam (systemically)	reimbursed
M01AC02	Tenoxicam	reimbursed
M03BX06	Mephenesin (oral)	non-reimbursed
M03BX06	Mephenesin (ointment)	non-approved
M03BA03	Methocarbamol	non-approved
P01BC01	Quinine	non-reimbursed
M05BX06	Romosozumab	reimbursed
M03BX05	Thiocolchicoside	non-reimbursed
	**Gastroenterology**	
A05AA04	Obeticholic acid	reimbursed
A03FA03	Domperidone	non-reimbursed
N05AD08	Droperidol	non-reimbursed
A04AD05	Metopimazine	non-approved
A02AD04	Hydrotalcite	non-approved
A07BC05	Diosmectite	non-reimbursed

© 2024 Fortinguerra F et al. *JAMA Network Open.*

A07BC02	Kaolin (alone or combined)	non-approved
A02AX	Beidellitic montmorillonite (monmectite)	non-approved
A06AX05	Prucalopride	non-reimbursed
A07DA02	Opium tincture	non-approved
C05AE01	Glyceryl trinitrate (0.4% ointment)	non-reimbursed
	**Gynaecology, Endocrinology**	
G03CX01	Tibolone	reimbursed
G03XB02	Ulipristal 5 mg	reimbursed
	**Infectious diseases**	
J01MA14	Moxifloxacin	reimbursed
	**Neurology, Alzheimer's disease**	
N06DA02	Donepezil	reimbursed
N06DA04	Galantamine	reimbursed
N06DA03	Rivastigmine	reimbursed
N06DX01	Memantine	reimbursed
	**Neurology, Multiple sclerosis**	
L04AA34	Alemtuzumab	reimbursed
L04AA23	Natalizumab	reimbursed
	**Neurology**	
N06BX03	Piracetam	non-reimbursed
N03AX26	Fenfluramine	reimbursed
N07CA03	Flunarizine	non-reimbursed
N02CX06	Oxetorone	non-approved
N04BX01	Tolcapone	reimbursed
N06DX02	Ginkgo biloba	non reimbursed (parapharmaceutical product)
C04AX21	Naftidrofuryl	non-reimbursed
	**Pulmonology, Ear, Nose, and Throat (ENT)**	
R05X	Alpha-amylase	non-approved
R05CB06	Ambroxol	non-reimbursed
R05CB02	Bromhexine	non-reimbursed
R01AA3	Ephedrine	non-approved
R01AA08	Naphazoline	non-reimbursed
R01AA05	Oxymetazoline	non-reimbursed
R01AA04	Phenylephrine	non-reimbursed
R01BA02	Pseudoephedrine	non-approved
R01AB08	Tuaminoheptane	non-reimbursed
R01AA07	Xylométazoline	non-reimbursed
R05CB16	Mannitol (inhalation powder)	non-reimbursed
L01EX09	Nintedanib[b]	reimbursed
R06AD08	Oxomemazine	non-approved
R05DB05	Pentoxyverine	non-reimbursed (over the counter medicine)
R05DA08	Pholcodine	non-approved
R03DX07	Roflumilast	reimbursed
R01AD07	Tixocortol (mouth spray)	non-approved
	**Psychiatry, Addiction**	
N06AX22	Agomelatine	non-reimbursed
N06AB04	Citalopram	reimbursed
N06AB10	Escitalopram	reimbursed
N06AX21	Duloxetine	reimbursed
N06AX16	Venlafaxine	reimbursed
N06AX17	Milnacipran	non-approved
N06AX27	Esketamine (nasal spray)	reimbursed
N05BX03	Etifoxine	non-approved
G04BX14	Dapoxetine	non-reimbursed
N06AX14	Tianeptine	non-approved
	**Smoking cessation**	
N06AX12	Bupropione	non-reimbursed
	**Urology**	
G04BX15	Pentosan polysulfate (oral)	non-reimbursed

[a] Drugs in red were not retrieved in the Italian administrative pharmaceutical databases because non-approved (n=20), non-reimbursed (n=6), non-commercialized (n=3) or not considered as a drug (n=1) in Italy.

[b] Nintendanib is mentioned twice in the *Prescrire*'s list of "Drugs to avoid", in lung cancer (ATC L) and idiopathic pulmonary fibrosis (ATC R), but it has been counted as one drug in the analysis.

© 2024 Fortinguerra F et al. *JAMA Network Open.*
