# Supplementary material for: Pharmaceutical Expenditure and Consumption of Recommended Drugs to Avoid in Italy
Source: JAMA Netw Open. 2024 Nov 20;7(11):e2446237. doi: 10.1001/jamanetworkopen.2024.46237 (PMC11579788; doi:10.1001/jamanetworkopen.2024.46237)
Supplement: Supplement 2. — Data Sharing Statement [file jamanetwopen-e2446237-s002.pdf]

Data Sharing Statement

Fortinguerra. Pharmaceutical Expenditure and Consumption of Recommended Drugs to Avoid in Italy. *JAMA Netw Open*. Published November 20, 2024.
doi:10.1001/jamanetworkopen.2024.46237

Data
Data available: No
